# Limbic progesterone receptors regulate spatial memory

**DOI:** 10.1038/s41598-023-29100-2

**Published:** 2023-02-07

**Authors:** Suchitra Joshi, Cedric L. Williams, Jaideep Kapur

**Affiliations:** 1 Department of Neurology, University of Virginia, Health Sciences Center, P.O. Box 801330, Charlottesville, VA 22908 USA; 2 Department of Psychology, University of Virginia, Charlottesville, VA 22908 USA; 3 Department of Neuroscience, University of Virginia, Charlottesville, VA 22908 USA; 4 UVA Brain Institute, University of Virginia, Charlottesville, VA 22908 USA

**Keywords:** Neuroscience, Physiology

## Abstract

Progesterone and its receptors (PRs) participate in mating and reproduction, but their role in spatial declarative memory is not understood. Male mice expressed PRs, predominately in excitatory neurons, in brain regions that support spatial memory, such as the hippocampus and entorhinal cortex (EC). Furthermore, segesterone, a specific PR agonist, activates neurons in both the EC and hippocampus. We assessed the contribution of PRs in promoting spatial and non-spatial cognitive learning in male mice by examining the performance of mice lacking this receptor (PRKO), in novel object recognition, object placement, Y-maze alternation, and Morris-Water Maze (MWM) tasks. In the recognition test, the PRKO mice preferred the familiar object over the novel object. A similar preference for the familiar object was also seen following the EC-specific deletion of PRs. PRKO mice were also unable to recognize the change in object position. We confirmed deficits in spatial memory of PRKO mice by testing them on the Y-maze forced alternation and MWM tasks; PR deletion affected animal’s performance in both these tasks. In contrast to spatial tasks, PR removal did not alter the response to fear conditioning. These studies provide novel insights into the role of PRs in facilitating spatial, declarative memory in males, which may help with finding reproductive partners.

## Introduction

Progesterone, a critical neuromodulator, regulates reproductive and non-reproductive behaviors in females. It regulates lordosis through mechanisms dependent on progesterone receptors (PRs)^1–4^. It also regulates spatial and conditioned learning, motivated and rewarding, as well as depressive behaviors in females (reviewed in^5^). The studies evaluating progesterone’s effects on various non-reproductive behaviors have evaluated rapid-onset, transient effects, which were likely mediated by allopregnanolone and related neurosteroid metabolic products of progesterone^6,7^. Not surprisingly, these progesterone actions are present in PR knockout mice^6,7^. PRs exert neuronal effects through gene expression, with relatively slow emergence^8–10^. Thus, the prior studies did not test delayed and more prolonged progesterone effects on cognitive behavior, which the current study explores with PRs.

Progesterone is also present in males who display progesterone levels similar to the concentrations detected in postmenopausal women^11^. In rodents, newborn males and females have comparable levels of circulating progesterone, whereas, in adults, the levels are lower in males than those in females^12,13^. The brain also synthesizes progesterone and other neurosteroids from cholesterol, and in male mice the brain progesterone levels are comparable to those in females in the diestrus stage^12^, and remain stable. PR expression is stronger in males than in females during fetal and neonatal period^14,15^. Thus, males are more sensitive to progesterone’s neuromodulatory effects during a short developmental window. Testosterone regulates the PR expression in neonatal male brain via conversion to estrogen and activation of estrogen receptors^16^. The progesterone-PR signaling plays a critical role in sexual dimorphic differentiation of the brain during development, and its blockade affects male sexual behavior in adulthood^14,17,18^. Furthermore, in the absence of testosterone, progesterone facilitates male sexual behavior through PR-regulated signaling^18–21^. Male mice lacking PRs have enhanced mating behavior and are less aggressive to intruder male or newborn pups^20,21^. Thus PR activity during development regulates male-typical behaviors and masculinization of the brain.

PR activity in neonatal males is also critical for the maturation of prefrontal cortex and the development of mesocortical and hippocampal circuitry, blocking PR activity during development results in lasting alterations in object recognition and cognitive flexibility (ability to learn new rules)^22,23^. This suggests that progesterone-PR signaling may also be critical for non-reproductive behaviors in males. However, in contrast to a thorough characterization of PR expression in neonatal male brain^15^, their distribution in the limbic brain regions is poorly described in adult males. Additionally, whether PRs are functional and regulate the activity of the hippocampus and entorhinal cortex, which participate in spatial and episodic memories, in adult males is also unclear.

Three separate approaches were employed to address each of these possibilities. We first revealed the contribution of PRs in promoting cognitive processing in male mice by examining the consequences of PR knockout (PRKO) on a battery of short and long-term memory tests. The performance of PRKO and wild-type mice were evaluated in learning tasks that included novel object recognition, object place recognition, Y-maze alternation, and Morris Water Maze learning assays. The involvement of PRs in regulating emotional memory was assessed with a standard contextual fear conditioning test. Our second approach included the use of qRT-PCR to validate the specificity of PR mRNA expression across limbic regions encompassing the entorhinal cortex (EC) and hippocampus. The final approach involved detecting the cellular distribution of PRs within discrete subnuclei of the EC and hippocampus. This was accomplished by using male mice expressing a Cre recombinase under the regulation of *Pgr* promoter to label PR-expressing cells with the fluorescent label, tdTomato. In summary, the combined experiments examined the potential contributions and distribution of PRs across the EC and hippocampus that may regulate spatial and emotional memory.

## Results

### EC and hippocampus of adult male mice express PRs

**Figure 1:**
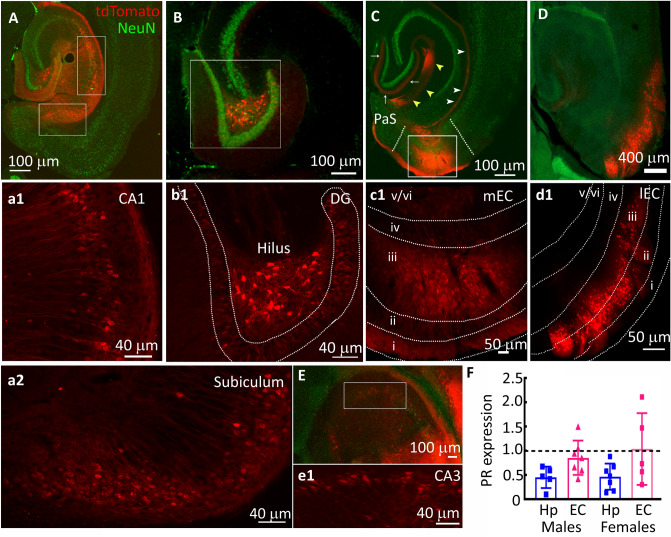
PR expression in the EC and hippocampus of adult male mice. (**A**–**E**) Images from representative Pgr-Cre animals injected with AAV9 expressing a flexed-tdTomato. The AAVs were injected into the hippocampus and EC. The sections were counterstained with a neuronal marker protein NeuN (Green) to label neurons. The tdTomato labeled terminals of the perforant path (white arrows), temporoammonic pathway (white arrowheads), and temporoammonic Alvear path fibers (yellow arrowheads) are also marked in 1C. (**a1**, **a2**, **b1**–**e1**) Magnified boxed regions from A-E showing neuronal tdTomato expression. (**F**) PR mRNA expression in the EC and hippocampus (Hp) of male mice, n = 7 for EC and 5 for Hp. The PR mRNA expression (1.12 ± 0.29, n = 7) in the uterine tissue isolated from females in the estrus stage of the cycle was used for normalization (dotted black line). The expression of PR mRNA in the EC and Hp tissue isolated from female mice in the estrus stage is shown for comparison (n = 5 EC and 7 Hp).

Previous studies described PR expression in the hypothalamic nuclei of male mice but not in the limbic regions. Furthermore, no quantitative comparison of the PR expression in the EC and hippocampus of male and female animals is available. We evaluated PR mRNA expression in the EC and hippocampal tissue using a qRT-PCR assay. PR mRNA was present in the EC and hippocampi of male mice (Fig. 1F). Their levels were comparable to those of females in the estrus stage of the cycle *t*(10) = 0.0964, *p* = 0.92 male versus female hippocampus; *t*(10) = 0.568, *p* = 0.58 male vs female EC.

We then studied the cellular distribution of PRs in the EC and hippocampus of male mice. Because the commercial antibodies lacked the specificity necessary for immunohistochemical detection (Fig. 8D), we used mice expressing a Cre recombinase under the control of the *Pgr* promoter to label PR-expressing cells with tdTomato to define the cellular distribution of PRs.

The tdTomato expression was evident in the CA1 and subicular neurons (Fig. 1A,a1,a2). In the CA1 region, the labeled neurons were present in the stratum pyramidale and stratum oriens. The DGC tdTomato expression was weaker than that in the CA1 and subiculum (Fig. 1B,b1). The tdTomato expression in the CA3 region was also sparse (Fig. 1E). Several neurons in the dentate hilus also expressed tdTomato (Fig. 1B,b1). The neurons of all three EC subdivisions, medial (mEC), central (cEC), and lateral (lEC), also expressed tdTomato (Fig. 1C,D,c1,d1). The tdTomato-expressing cells were commonly present within the central EC in layer III. On the other hand, the mEC and lEC tdTomato expression was spread in layers II/III. The layer II/III EC neurons project to the DGCs and CA1 neurons via the perforant pathway (PP) and temporo-ammonic pathway (TA), respectively; the terminals of the labeled EC neurons marked the molecular layer of the dentate gyrus (Fig. 1C). There was tdTomato fluorescence in the temporo-ammonic Alvear path fibers and stratum lacunosum moleculare of the CA1 hippocampus (Fig. 1C). The EC-expressing tdTomato fluorescence normalized to that of NeuN was 42.3 ± 23.09 (n = 10 sections from 5 animals) (mean ± SD, here and subsequently). That in the CA1 + subiculum was 26.34 ± 10.56 (n = 8 sections from 3 animals). The tdTomato fluorescence was the least in the DG, 10.56 ± 7.66 (n = 7 sections from 3 animals). The EC and CA1 PR expression was similar (*F*(*2*, *22*) = *7.793*, *p* = 0.123 ordinary one-way ANOVA with post hoc Tukey’s multiple comparisons); however, the expression in the DG was substantially lower than that in the EC (*F*(*2*, *22*) = *7.793*, *p* = 0.002 ordinary one-way ANOVA with post hoc Tukey’s multiple comparisons).

**Figure 2:**
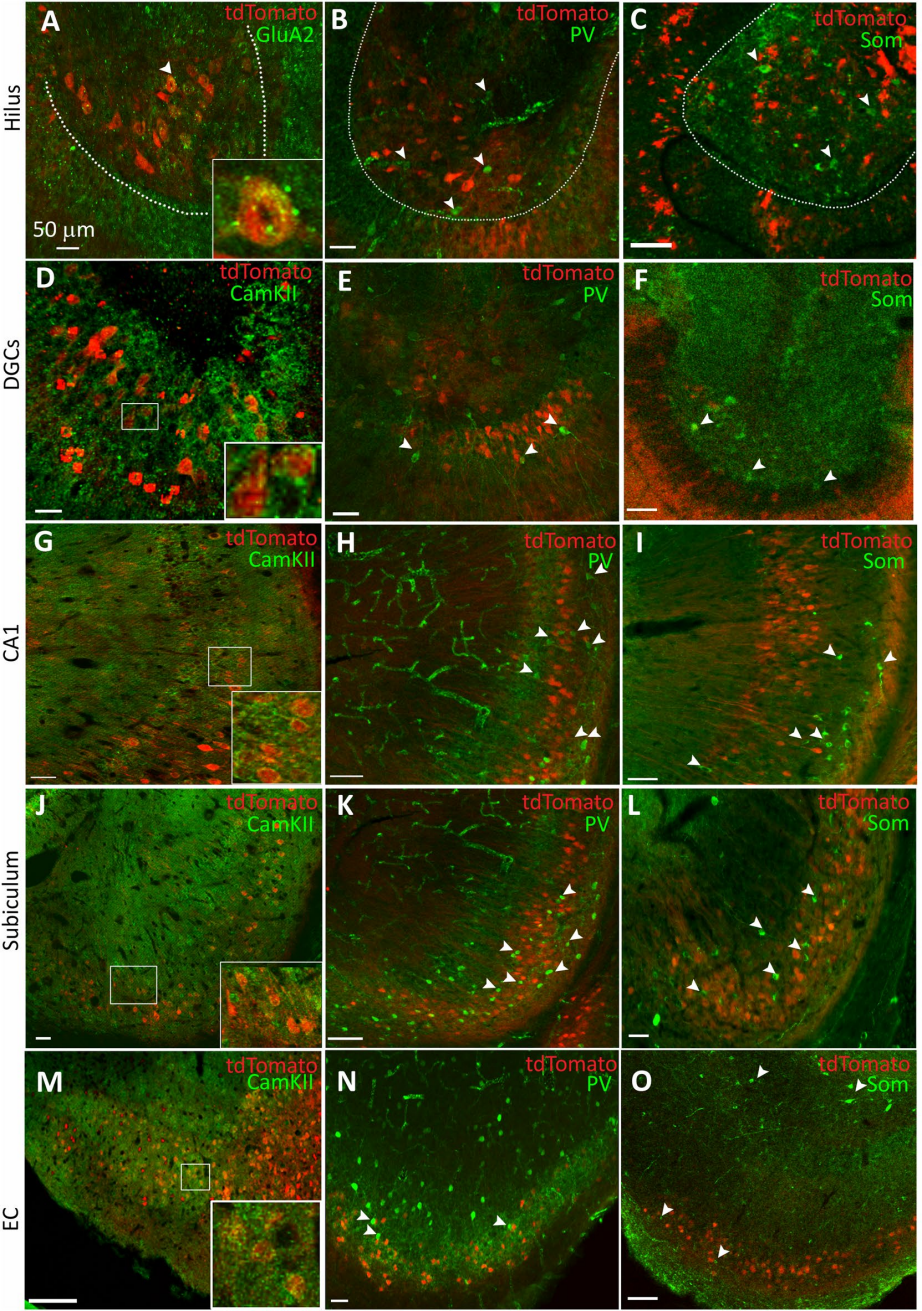
PR expression in the principal neurons vs interneurons. (**A**–**C**) Representative images of the dentate hilus of Pgr-Cre mice injected with AAV expressing flex tdTomato. The images show colocalization of tdTomato expression with that of the immunoreactivity of the GluA2 subunits of AMPA receptors, parvalbumin (PV), and somatostatin (Som) neuropeptides respectively. The expression of the GluA2 subunit marked the hilar mossy cells, whereas, parvalbumin and somatostatin labeled two types of interneurons. The scale bars in these and all other images in this figure correspond to 50 μm. The hilar neuron marked by an arrow is magnified in the inset in (**A**) to show punctate GluA2 subunit immunoreactivity in a tdTomato-expressing neuron. The arrow heads in (**B**, **C**) mark the parvalbumin and somatostatin-expressing neurons respectively, none of which colocalized with tdTomato fluorescence. (**D**–**F**) Representative images showing colocalization of tdTomato with that of CamKII, PV, and Som respectively in the DGCs. The inset corresponds to the boxed region and shows perisomatic CamKII immunoreactivity in the tdTomato-positive DGCs. The arrows in (**E**, **F**) point to the interneurons. (**G**–**I**) Colocalization of tdTomato and CamKII, PV, and Som in the CA1. The boxed region in (**G**) is magnified in the inset, arrows in (**H**, **I**) point the interneurons. (**J**–**L**) Colocalization of tdTomato with CamKII, PV, and Som expression respectively. The boxed region in (**J**) is magnified as an inset, arrows in (**K**, **L**) point to the interneurons. (**M**–**O**) Colocalization of tdTomato in the medial EC with CamKII, PV, and Som respectively. Please note that som labeling in the superficial layers of EC was sparse, but som-positive neurons were present in the deeper layers (marked by arrows). Also please note that the number of som-expressing neurons seems to be substantially lower than those expressing PV.

Principal neurons preferentially expressed PRs. We evaluated tdTomato colocalization with CamKII protein, a marker of glutamatergic neurons. The tdTomato labeled DGCs, CA1, subicular, and EC neurons colocalized with CamKII (Fig. 2D,G,J,M). We evaluated tdTomato fluorescence colocalization with parvalbumin (PV) and somatostatin (Som) peptides to evaluate PR expression in the interneurons. Not many tdTomato-expressing neurons expressed PV in the DG cell layer, CA1, subiculum, and EC (Fig. 2B,E,H,K,N). The tdTomato colocalization with the Som immunoreactivity was also sparse. Only a few neurons in the hippocampus and EC expressed Som (Fig. 2C,F,I,L,O). To confirm that PRs were primarily expressed in the glutamatergic neurons, the percentage of tdTomato-expressing neurons that expressed CamKII or PV or Som was determined and compared (Table 1). The colocalization of PRs with PV or Som immunoreactivity was observed in only a few sections, and most PR-expressing neurons in the CA1 + subiculum expressed CamKII as compared to PV or Som (*F*(*2*, *23*) = *2275*, *p* < 0.0001, ordinary one-way ANOVA post-hoc comparisons PV vs CamKII and som vs CamKII). Similarly, the CamKII colocalization with tdTomato was also higher in the EC (*F*(*2*, *12*) = *293.5*, *p* < 0.0001, ordinary one-way ANOVA post-hoc comparisons PV vs. CamKII and som vs. CamKII) and in the DG (*F*(*2*, *19*) = *688*, *p* < 0.0001, ordinary one-way ANOVA post-hoc comparisons PV vs CamKII and som vs CamKII). The number of PV or Som-expressing neurons that also expressed tdTomato did not differ in the DG (*t*(13) = 1.618, *p* = 0.1297, student’s t-test) or EC (*t*(6) = 0.2558, *p* = 0.8066, student’s t-test); however more PV-expressing neurons than som-expressing neurons were tdTomato labeled in CA1 (*t*(14) = 3.295, *p* = 0.0053, student’s t-test).

**Table 1:** Quantification of colocalization of tdTomato fluorescence with glutamatergic neuronal marker CamKII and GABAergic neuronal markers PV and Som.

Marker	CA1 + subiculcum	DG	EC	Hilus
CamKII	89.25 ± 5.8 n = 10 slices from 3 animals	88.31 ± 8.8 n = 7 slices from 2 animals	83.72 ± 7.3 n = 7 slices/3 animals	
PV	2.314 ± 2.345 n = 4 slices from 2 animals	1.479 ± 2.407 n = 9 slices from 3 animals	1.240 ± 1.687 n = 4 slices from 2 animals	3.789 ± 5.579 n = 9 slices from 3 animals
Som	0.1244 ± 0.4309 n = 12 slices from 5 animals	0 ± 0 n = 7 slices from 5 animals	1.002 ± 0.7904 n = 4 slices from 2 animals	19.05 ± 19.49 n = 15 slices from 4 animals
GluA2				26.96 ± 18.15 n = 8 slices from 2 animals

The values represent Mean ± SD of the percent tdTomato-expressing neurons that colocalized with the marker protein.

There were tdTomato-expressing cells in the dentate hilus, a region populated by inhibitory GABAergic interneurons and excitatory mossy cells^24^. The expression of GluA2/3 subunits of AMPA receptors distinguishes mossy cells from interneurons^25^. Thus, to evaluate whether the tdTomato-expressing hilar neurons were mossy cells, we determined their colocalization with the GluA2 subunit protein. Many tdTomato-expressing hilar neurons also expressed punctate and perisomatic GluA2 immunoreactivity (Fig. 2A). We tested whether the hilar tdTomato-positive neurons expressed PV or Som neuropeptides. As expected, very few hilar neurons expressing PV or Som expressed tdTomato (Fig. 2B,C). We also compared the colocalization of tdTomato expression with that of GluA2, PV, or Som (Table 1). The hilar tdTomato-expressing neurons expressed GluA2 subunit or Som, but not PV (*F*(*2*, *28*) = *4.02*, *p* = 0.029, ordinary one-way ANOVA post-hoc Tukey’s comparison PV vs GluA2).

Thus, with the hippocampus and EC PRs were expressed in the principal neurons and in only a small fraction of inhibitory interneurons. We then tested the effect of activating PRs in EC and the hippocampus.

### PRs activate neurons in the EC cortex and hippocampi of male mice

It is established that PRs increase EC neuronal excitability in female rats^26^. We therefore expected to observe a similar effect of PR on neuronal activity in males. The activity-reporter targeted recombination in active populations-2 (TRAP2) mice^27^ were treated with segesterone or saline in their home cage to test the effect of activating PRs.

**Figure 3:**
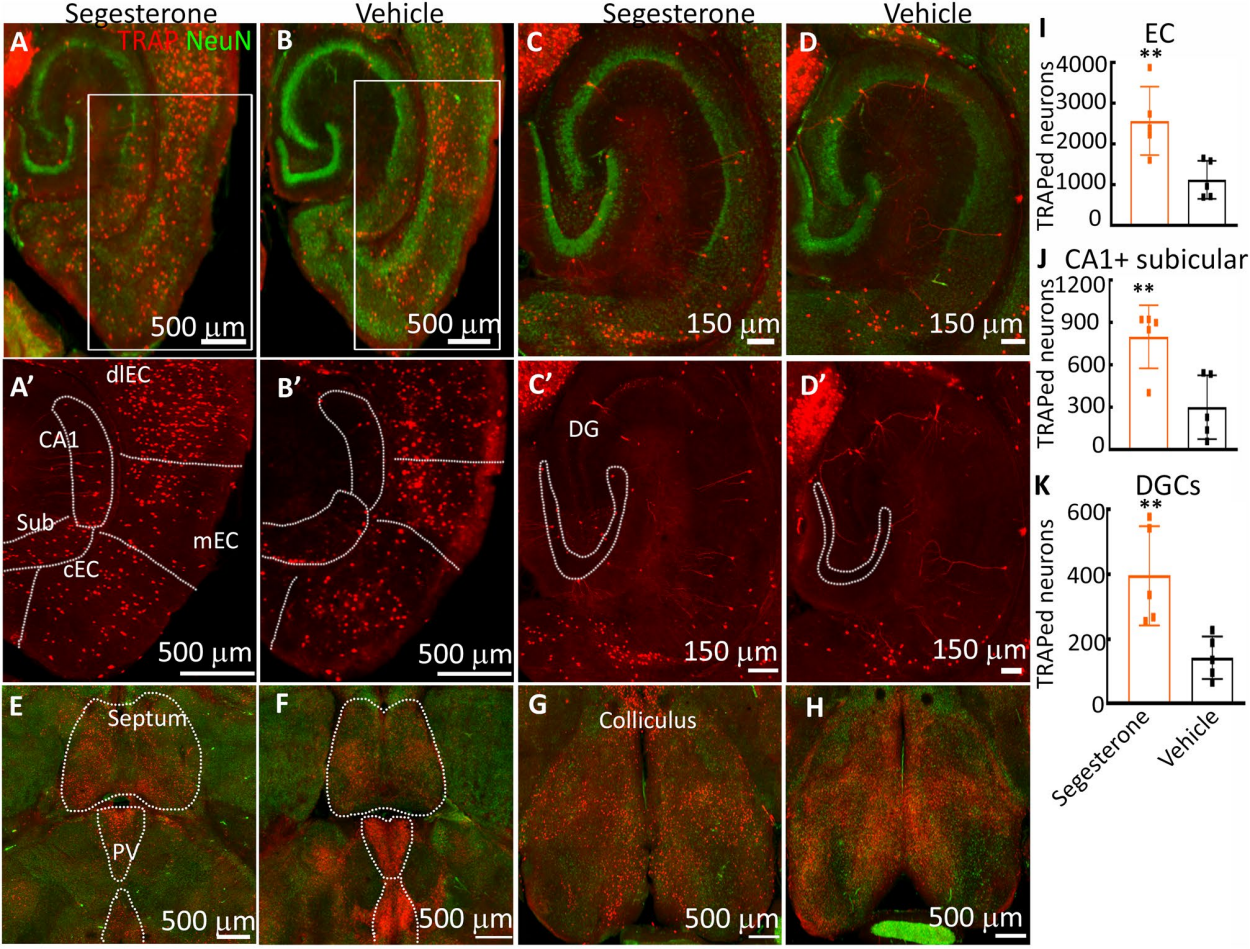
The effect of PR activation on the number of active neurons in the EC and hippocampus of adult male mice. (**A**, **B**) Representative images from segesterone- (10 mg/kg, subcutaneous) and vehicle- (20% β-hydroxycyclodextrin) treated male mice respectively. 4OHT was administered 2 days after the treatment in home cages to TRAP the active neurons (red). The green fluorescence corresponds to the expression of neuronal marker protein NeuN. (**A′**, **B′**) Magnified boxed areas from A and B respectively. The CA1, subiculum (sub), central (cEC), medial (mEC) and dorsolateral (dlEC) EC regions are marked. (**C**, **D**) Representative images from segesterone- and vehicle-treated mice show active DGCs, the NeuN immunoreactivity is shown in green. (**C′**, **D′**) Images showing the TRAPed DGCs in the segesterone- and vehicle-treated animals. (**E**, **G**) and (**F**, **H**) Images showing the TRAPed neurons in the septum, paraventricular thalamic nucleus, and colliculus of segesterone- and vehicle-treated animals respectively. (**I**–**K**) The number of TRAPed neurons in the entorhinal cortex, combined CA1 and subiculum, and DGCs in segesterone- and vehicle-treated animals, n = 5 each, **p = 0.0098 for EC, *p* = 0.008 for CA1 and subiculum, and *p* = 0.0094 for DGCs, student’s t-test.

Segesterone treatment enhanced EC neuronal activity as evidenced by the number of tdTomato-expressing neurons recorded in the EC of vehicle, versus segesterone-treated animals (Fig. 3A,B); however, in the segesterone-treated animals, there were many more labeled neurons than in the vehicle-treated controls. The labeled neurons were present in all the subfields of the EC: central, medial, and lateral. Within the medial EC, the labeled neurons were concentrated in layers II/III and V, whereas, in the lateral EC, they were present in all the layers. Like in EC, segesterone treatment also activated hippocampal neurons. Neurons in the DG, CA1 and subiculum expressed tdTomato (Fig. 3). Although PR-expressing neurons were present in the dentate hilus, the tdTomato-expressing neurons were sparse in this region (Fig. 3C,D). We counted all the labeled neurons in both groups within EC and CA1 + subicular regions and DGCs. Segesterone treatment activated twice as many EC neurons (Fig. 3I, n = 5, *t*(*8*) = 3.37, *p* = 0.0098, student’s t-test), and CA1, and subicular neurons as the vehicle-treated animals (Fig. 3J, n = 5, *t*(*8*) = 3.508, *p* = 0.008, student’s t-test). The number of active DGCs in segesterone-treated animals was thrice that of the vehicle-treated animals (Fig. 3K, n = 5, *t*(*8*) = 3.394, *p* = 0.0094, student’s t-test). Segesterone treatment did not activate lateral septum or collicular neurons. The number of TRAPed neurons in the lateral septum and colliculus was similar in the segesterone and saline-treated animals (Fig. 3E–H), suggesting a specific segesterone effect. Thus, segesterone activated PR-expressing neurons or their downstream targets.

### Blockade of PRs impacted object recognition in male mice

**Figure 4:**
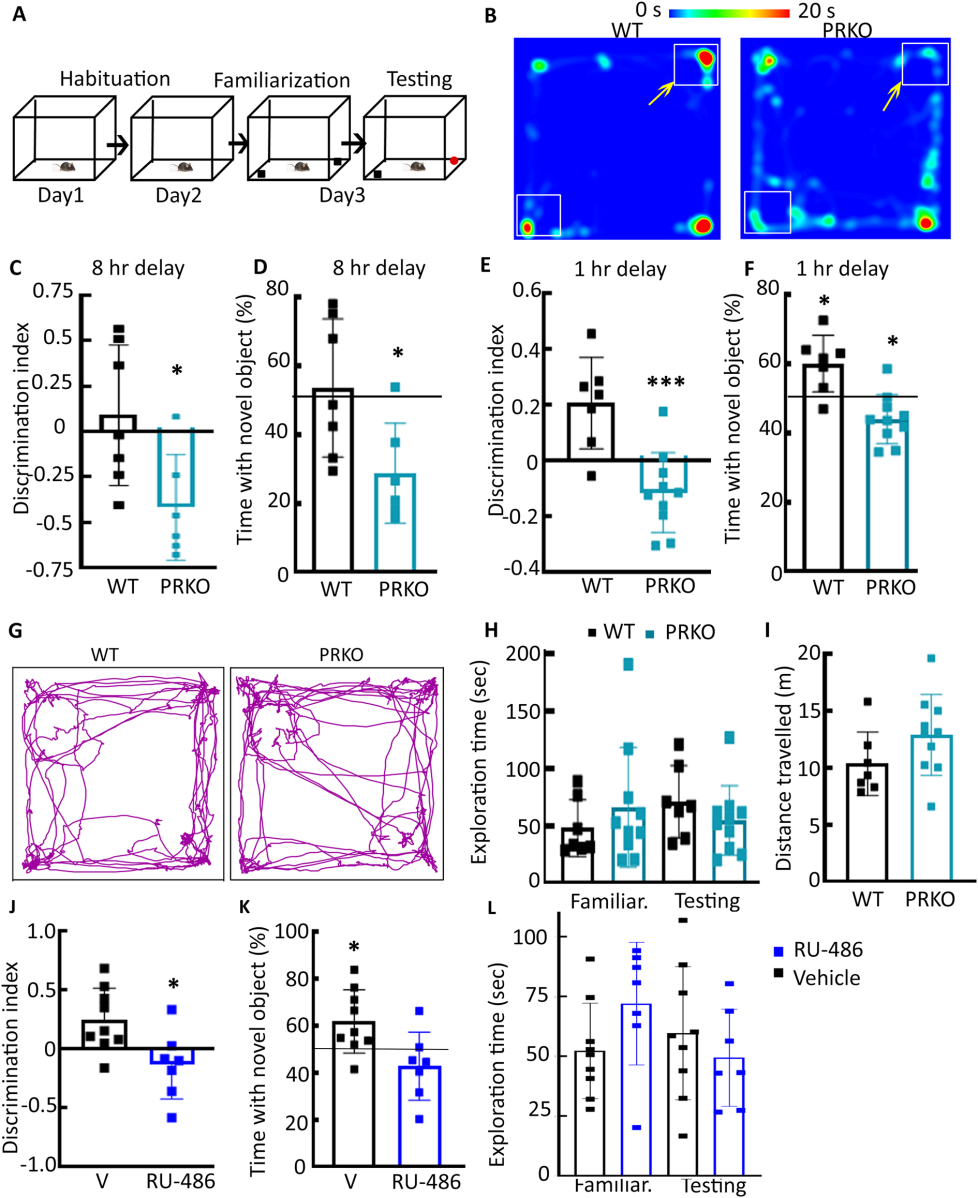
The effect of PR deletion on novel object recognition in adult male mice. (**A**) A schematic showing habituation, familiarization, and testing of animals in a novel object recognition test. The assay was performed as described in the methods section. (**B**) A heat map of the time spent by representative PR knockout (PRKO) and littermate wildtype (WT) mice during the testing phase performed 8 h after the familiarization. The yellow arrows denote the position of novel objects. (**C**) Discrimination index in the WT and PRKO mice, n = 7 WT and 6 PRKO, *p = 0.023, student’s t-test. (**D**) The average and SD of the percent novel object exploration time evaluated after a delay of 8 h, n = 7 WT and 6 PRKO, *p = 0.0167, one-sample t-test. The black line denotes 50%, corresponding to chance performance. (**E**) Discrimination index in the WT and PRKO mice, ***p = 0.0006, student’s t-test. (**F**) The percent novel object exploration time evaluated after one-hour delay, n = 7 WT and 10 PRKO, *p = 0.0106, one sample t-test for WT and *p* = 0.0311, one sample t-test for PRKO. The black line denotes 50%, which is chance performance. (**G**) Movement of representative WT and PRKO mice in the open-field arena. (**H**) Mean and standard deviation of the total exploration time in the WT and PRKO mice during familiarization and (1-h delay) testing phases. (**I**) Mean and standard deviation of the total distance traveled by 7 WT and 10 PRKO mice. (**J**) Discrimination index in the vehicle- and RU-486-treated mice, *p = 0.016, student’s t-test. The animals were treated daily with vehicle (20% β-hydroxycyclodextrin daily subcutaneous, sc) and RU-486 (10 mg/kg, sc) for a week. The experiment was performed a day after the last injection. There was a one-hour delay between the familiarization and testing phases. (**K**) The percentage novel object exploration of the vehicle- and RU-486-treated animals, n = 9 vehicle-treated and 7 RU-486-treated, *p = 0.026, one sample t-test for WT mice. The black line denotes 50%, corresponding to performance by chance. (**L**) Average and standard deviation of the total exploration time in the WT and PRKO mice during familiarization and testing phases.

We further tested the role of PRs in EC and the hippocampus, which critically regulate spatial memory^28–31^, using mice lacking PR expression in the brain (PR^fl/fl^-Cre+ve, PRKO). Littermate PR^fl/fl^-Cre−ve mice were used as controls (referred to as WT). Using an object recognition task, we compared declarative memory in male mice lacking PRs with WT controls (Fig. 4A). WT mice that were returned to the testing apparatus 8 h after the familiarization phase, spent more time exploring the novel object than the PRKO mice (Fig. 4B , n = 6 PRKO and 7 WT). The discrimination index is a commonly used quantitative measure of short term memory since it calculates an animal’s capacity to distinguish new items from items they have previously encountered during the familiarization phase in the NOR task. There were distinct differences in short term memory accuracy between the PRKO and WT mice (Fig. 4C, *t*(*11*) = 2.633, *p* = 0.023, student’s t-test). The negative discrimination indices in all the PRKO mice indicated a preference for the familiar object. Since, the formation of a proper memory trace for the familiar object should lead to the exploration of novel object above the level of chance (50%)^32^, we determined whether the novel object exploration time in the WT mice crossed the chance threshold (Fig. 4D). However, the novel object exploration time in WT mice was similar to the chance exploration (*t*(*6*) = 0.4968, *p* = 0.64, one sample t-test). Furthermore, that in the PRKO mice was substantially lower than the 50% threshold (*t*(*5*) = 3.53, *p* = 0.0167, one sample t-test). This indicated that the PRKO mice had a preference for familiar object. However, even in the WT mice, memory trace encoding, consolidation or retrieval did not occur.

We evaluated whether a shorter delay could improve object recognition in the WT mice compared with the PRKO (n = 10 PRKO, 7 WT). Following an hour delay, WT mice explored the novel object for longer periods than the PRKO mice (Fig. 4F), and this exploration time was greater than chance (Fig. 4F, *t*(*6*) = 3.317, *p* = 0.0161, one sample t-test). However, the novel object preference of the PRKO mice remained below the 50% threshold (*t*(*9*) = 2.553, *p* = 0.0311, one sample t-test). The discrimination indices were also significantly different between the WT and PRKO mice (Fig. 4E, *t*(*15*) = 4.291, *p* = 0.0006, student’s t-test). Thus, WT mice preferred the novel object whereas PRKO mice preferred the familiar object.

We determined whether PR deletion alters exploratory activity or locomotion. However, the two genotypes spent the same amount of time exploring objects during the familiarization and testing phases (Fig. 4H, *F*(*3,30*) = *0.5892*, *p* = 0.6268, ordinary one-way ANOVA). They traveled comparable distances (Fig. 4G,I, *t*(*15*) = 1.569, *p* = 0.1375, student’s t-test). Thus, PR removal did not affect object exploration or locomotion.

This deficit in PRKO mice was not due to the absence of progesterone. Progesterone levels in the combined EC and hippocampal tissue lysates measured using an ELISA assay were also similar between the WT males: 20.5 ± 5.7 pg/mg, n = 6, and PRKO males: 21.1 ± 9.7 pg/mg, n = 5, (*t*(*9*) = 0.1364, *p* = 0.89, student’s t-test).

### The pharmacological blockade of PRs also affected novel object recognition

An absence of PRs throughout the development of the PRKO mice may have contributed to the observed deficits in adult mice. We tested whether blocking the PR activity for a short period in adult mice also impacted object recognition (n = 7 RU-486-treated and 9 vehicle-treated). Like the PRKO mice, the discrimination index in the RU-486-treated animals was negative and distinct from that in the vehicle-treated mice (Fig. 4J, *t*(*14*) = 2.73, *p* = 0.016, student’s t-test). Furthermore, the novel object exploration was above chance performance in vehicle-treated mice (Fig. 4K, *t*(*8*) = 2.725, *p* = 0.026, one sample t-test), and at a chance level of performance in RU-486-treated mice (*t*(*6*) = 1.264, *p* = 0.253, one sample t-test). Thus, RU-486-treated mice were impaired in novel object recognition and did not have a preference for either of the objects. The vehicle- and RU-486-treated groups were comparable in the time spent exploring the two objects during both the familiarization and testing phases (Fig. 4L, *F*(*3,28*) = *1.311*, *p* = 0.29, ordinary one-way ANOVA). Despite the similarity in object exploration, the lack of preference for the novel object during the testing phase indicates that the pharmacological blockade of PRs in adulthood interferes with short-term memory processes necessary to detect environmental changes such as that presented during the novelty test.

### Male PRKO mice could also not recognize object place change

**Figure 5:**
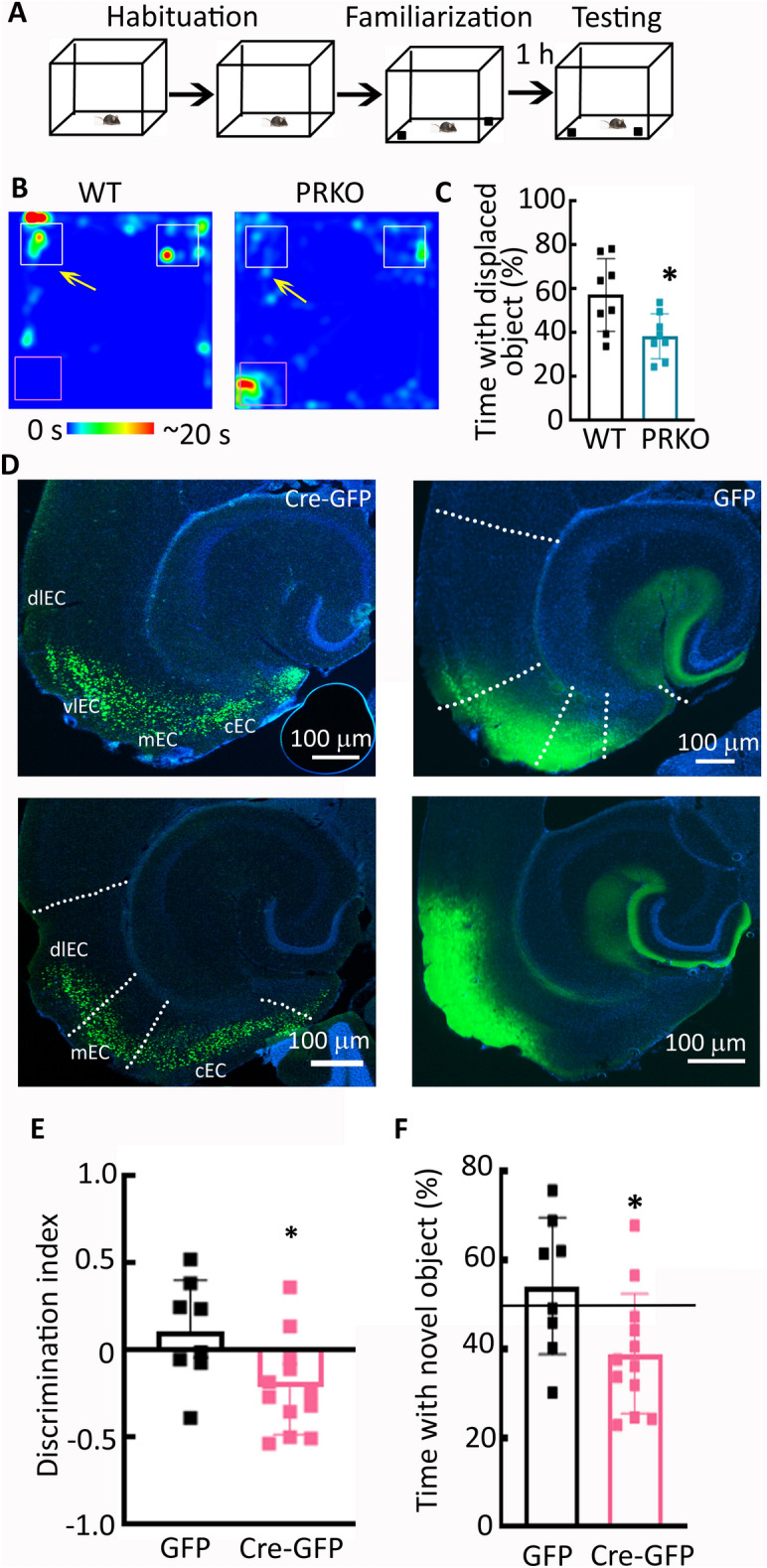
The effect of PR deletion on object place recognition and EC-specific deletion on novel object recognition. (**A**) The schematic showing object place recognition testing. (**B**) Heat map from representative WT and PRKO mice during the testing phase. The arrows mark the displaced object; purple squares mark their original position. The animals were tested after an hour. (**C**) The percent time spent in exploring the displaced object, n = 8 each, *p = 0.016 student’s t-test. (**D**) Images from representative animals injected with AAVs to express CamKII-driven Cre-GFP or GFP alone. Sections from the ventral and dorsal EC cortex are shown, the green fluorescence corresponds to Cre-GFP or GFP expression, and DAPI (blue) was used as a counterstain. (**E**) Discrimination indices in the Cre-GFP and GFP mice, *p = 0.0212, student’s t-test. (**F**) Percent novel object exploration in the animals injected with AAVs to express CamKII driven Cre-GFP or GFP alone, n = 8 for GFP alone and 12 for GFP-Cre, *p = 0.017 one sample t-test for Cre-GFP mice. The black line marks a chance performance 50%.

Episodic memories comprise an integration of the "what", "where," and “when” components^31,33^ of learned experiences and we found an impact of PR deletion or their pharmacological blockade on the "what" component. We evaluated whether PRs also regulated the "where" element, i.e. recognizing the object's place (Fig. 5A). The male PRKO mice also differed from the WT mice in recognizing the object's place change. The WT mice explored the displaced object more (Fig. 5B,C), but not the PRKO mice (Fig. 5B,C, n = 8 each, *t*(*14*) = 2.278, *p* = 0.016, student’s t-test). Together, these studies revealed that PR signaling was necessary for the integration of components that work collectively to form episodic-like memories.

### EC-specific deletion of PRs was sufficient to impair novel object recognition

The PRKO mice had impaired novel object recognition; however, these mice lacked the PRs in the entire brain. We evaluated whether the entorhinal cortex-specific deletion of PRs in adult mice affected object recognition. Expression of a GFP-tagged Cre recombinase in the EC cortex of adult males led to transduction of 70–80% of the entorhinal cortical neurons (Fig. 5D) and reduced the PR mRNA expression (45 ± 16%, n = 4). After confirming the EC-specific deletion of PRs, we tested the Cre-expressing animals in the novel object recognition task. The Cre-expressing mice did not show greater interest in the novel object (Fig. 5F). The novel object exploration time in the Cre-expressing animals was lower than the 50% threshold (Fig. 5F, n = 12, *t*(*11*) = 2.289, *p* = 0.017, one sample t-test), whereas the novel object exploration time in the GFP-expressing mice was at the threshold (n = 8, *t*(*7*) = 1.008, *p* = 0.347, one sample t-test). The discrimination index in the Cre-expressing mice was negative and distinct from that in the GFP-expressing mice (Fig. 5E, *t*(18) = 2.524, *p* = 0.0212, student’s t-test). Thus EC-specific deletion of PRs also impacted novel object recognition with the mice showing a preference for the familiar object.

### Spatial reference memory was affected in PRKO mice

**Figure 6:**
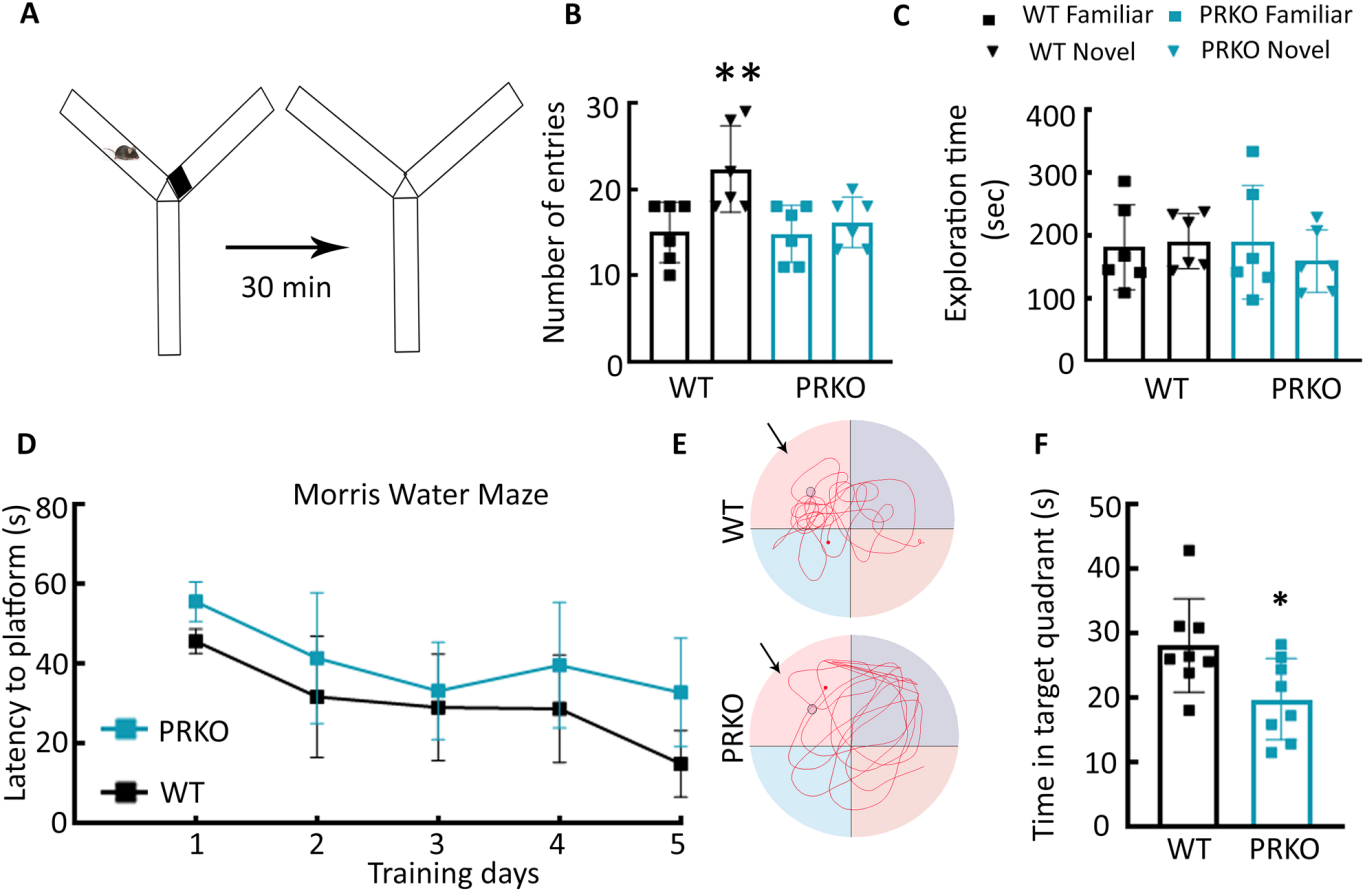
Effect of PR deletion on Y maze forced alternation and Morris Water Maze tasks. (**A**) A schematic of the Y maze forced-alternation test. (**B**) The number of entries in the novel arm by PRKO and WT males in a Y maze forced-alternation task, n = 6 each, *p = 0.0074, ANOVA with Sidák's multiple comparison WT familiar vs WT novel. (**C**) The time spent exploring the novel and familiar arms. (**D**) Latency to reach the platform during training, n = 8 each. (**E**) Track plots from representative WT and PRKO mice during the probe trial on day 6. The arrow marks the quadrant that held the escape platform during training sessions. Please note that the WT animal kept swimming in the region around the location of the platform (marked by the circle) during the probe trial whereas, the PRKO mouse swam in all the quadrants. (**F**) The time spent in the target quadrant during the probe trial, *p = 0.0277, student’s t-test.

We subsequently evaluated whether PR signaling also regulated other forms of memories. We confirmed that PRs regulate spatial memories using a Y-maze forced-alternation task^34^ (Fig. 6A). WT mice entered the novel arm more often when they were tested 30 min after the initial exposure to the Y-maze (Fig. 6B). The PRKO mice, in contrast, visited the familiar and novel arms equally (Fig. 6B, n = 6 each, *F*(*3,20*) = *5.306*, *p* = 0.0074, ordinary one-way ANOVA, post-hoc Sidák's multiple comparisons WT novel vs WT familiar). The time spent in the two arms did not differ in either genotype (Fig. 6C; *F*(*3,12*) = *0.211*, *p* = 0.8868, ordinary one-way ANOVA). The reduced number of entries in the novel arm by PRKO mice indicated that PR deletion impacted spatial reference memory.

To confirm the effects of PR deletion on spatial memory, we also tested the animals in a Morris water maze (MWM) task. During the 5 days of training, there was a gradual decrease in the latency to reach the escape platform in the WT and PRKO mice (Fig. 6D, n = 8 each, *F*(*4,60*) = *13.92*, *p* < *0.0001*, two-way repeated measures ANOVA). The acquisition rate was slower in the PRKO mice than in the WT mice (*F*(*1,14*) = *6.773*, *p* = 0.0209, two-way repeated measures ANOVA). Furthermore, on the day of testing, the WT mice spent longer in the target quadrant than the PRKO mice (Fig. 6E,F, *t*(14) = 2.556, *p* = 0.0277, student’s t-test). Thus spatial memory was affected in the PRKO mice.

### Contextual fear conditioning was not affected in the PRKO mice

**Figure 7:**
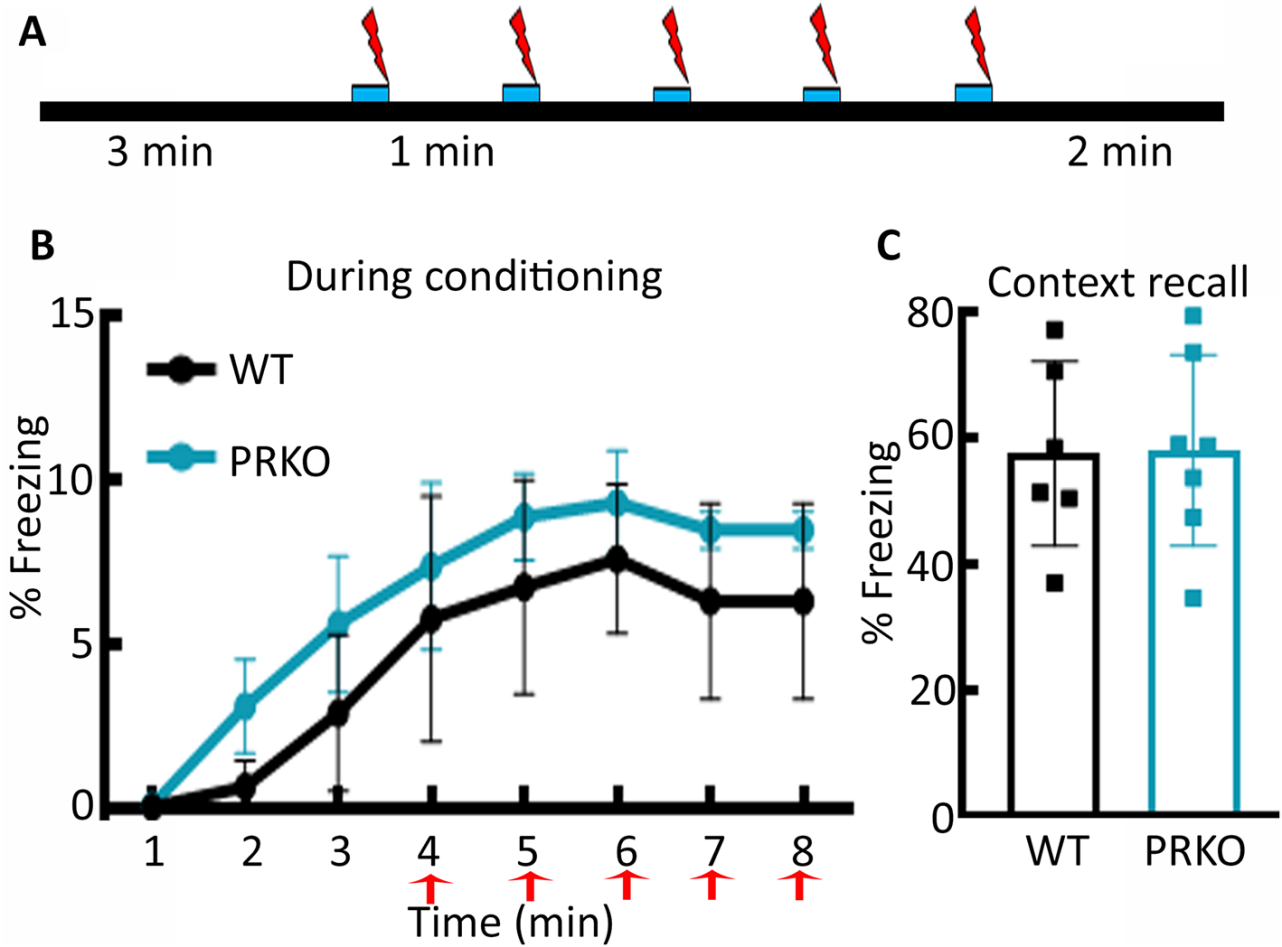
The cued-contextual fear conditioning in PRKO male mice. (**A**) A schematic showing fear conditioning and recall. Following an initial 3 min of chamber exploration, the animals received five 2-s footshocks that were contiguous with a 20-s tone. The animals were removed from the chamber two min after the last footshock. (**B**) Percent freezing in the WT and PRKO male mice following the five footshocks, n = 6 WT and 7 PRKO. (**C**) Percent freezing during the context recall was performed 24 h later. The number of replicates is the same as in E.

In contrast to spatial memories, implicit memories include procedural memories or those driven by stimulus or priming and do not involve a conscious recall of the events. We evaluated fear conditioning in WT and PRKO male mice to understand whether PR signaling also regulates implicit memories (Fig. 7A). Animals' immobility and freezing increased after each foot shock on the day of conditioning, and this was similar between the PRKO and WT mice (Fig. 7B, *F*(*1,10*) = *4.841*, *p* = 0.052, two-way repeated measures ANOVA). Freezing during the context recall testing performed 24 h later was identical in the two groups (Fig. 7C, *t*(9) = 0.7512, *p* = 0.47, student’s t-test). Thus, PR deletion did not impact contextual fear memory.

## Discussion

We demonstrate that principal neurons in the EC-hippocampal circuit express PRs, and ablating them causes spatial memory impairment. PR deletion affected spatial and declarative memories in male mice. First, PRs were expressed primarily in principal neurons within the EC and hippocampus of male mice. Activation of PRs with segesterone, increased the number of active neurons in the DGC, CA1, subicular, and EC. In contrast, disruption of PR signaling either with RU-486 or genetic ablation impaired short-term and long-term spatial memories but not fear conditioning. The PR-regulated spatial memory may offer reproductive advantages, helping animals find mates and food.

We found that PRs regulate spatial memory. The lateral and medial EC relay non-spatial and spatial information to the hippocampus, which integrates these inputs^35,36^. The hippocampal CA1 neurons (place cells) have tightly coupled firing to their fields^37^. The medial EC neurons (grid cells and border cells), particularly those in the superficial layers, also have strong space-firing fields^38–41^. The medial EC neurons receive sensory inputs from visual and parietal cortices through the post-rhinal cortex^42,43^. In contrast to the medial EC, the spatial firing selectivity of lateral EC neurons is weak^42^, and these neurons are proposed to function as object-responsive cells and object-trace cells^44^. Lesioning of the lateral EC affects recognition of object, its place, and context^45,46^. Both the medial and lateral EC neurons project to the DGCs via the perforant pathway, and lesions of the dentate gyrus also affect the recognition of objects' place^47^. This indicates that impairing EC-DG function impacts spatial memory. The potentiation of glutamatergic synaptic transmission termed long-term potentiation (LTP) is critical for memory formation; its blockade or saturation impairs cognitive processes^48,49^. The strong PR expression in the EC and hippocampi seen here and their role in regulating AMPA receptor-mediated transmission of these neurons found earlier^26,50^, raises the possibility that PR activity may regulate LTP in these neurons. Indeed, affected EC-DG communication is seen during aging and in Alzheimer’s disease (AD), conditions associated with cognitive decline. A loss of perforant path-DGC synapses in the outer molecular layer is seen in patients with mild cognitive impairment and early AD^51^. In contrast, aging modifies the lateral perforant path-DGC synapse plasticity^52^. A putative loss of PRs or PR-expressing neurons combined with declining progesterone levels could affect the normal transmission between EC-DG-CA1 neurons, which is critical for spatial memory.

The PRKO mice lacked PRs throughout development and may have affected maturation of the medial prefrontal cortex (mPFC) and hippocampal circuitry and impacted dopamine signaling, similar to that seen by developmental blockade of PR activity^22^. Since both these regions are critical for acquiring, storing, and retrieving memories, lack of PRs may induce a general cognitive impairment. Another study has found that developmental blockade of PRs also reduces cognitive flexibility and induces impulsivity^22^. Additionally, the preference of PRKO mice for the familiar object in the NOR task suggests a putative anxious phenotype. PR deletion may alter dopamine synthesis by ventral tegmental area neurons and affect the mesolimbic dopamine pathway^53^. Also, putative effects of PR deletion on progesterone’s metabolism to allopregnanolone, a potent anxiolytic agent, and/or changes in GABA-A receptor expression could also make the PRKO mice more anxious.

Prior studies have reported PR expression in the limbic regions, including the amygdala and hippocampus of the fetal brain^15,23^ and the adult female brain^26,54–57^. In contrast, extrahypothalamic PR expression in adult male brains is not characterized, although prior studies have suggested that PRs are expressed in the hippocampi^56,58^. We found that PR expression in the EC-hippocampal network was comparable between adult males and females. Furthermore, the PR-expressing cells in these regions also expressed CamKII, a marker of glutamatergic neurons, but far fewer number of inhibitory neurons expressed PRs, indicating their excitatory nature. Although we evaluated the colocalization of tdTomato with neuropeptides marking only two interneuron types, the high degree of colocalization with CamKII suggests that PR are not expressed in interneurons. The findings in dentate hilus, rich in interneurons, also support this as the PR-expressing cells mostly expressed GluA2 subunit, a marker for hilar mossy cells. A prior study in neonatal animals also did not find overlap between PR and GAD67 immunoreactivity in the dentate gyrus^23^. The PR expression in the EC has never been described before, whereas, the pattern of tdTomato expression in the hippocampus is similar to that described in females in a prior study^56^. Since the tdTomato expression is constitutive following cre-regulated recombination in the PR-expressing neurons, these studies do not provide a quantitative assessment of PR expression. However, the signal-to-noise ratio is high in this method as even a small amount of Cre is sufficient to permanently turn-on the tdTomato expression. Also, labeling of the PR-expressing neurons with tdTomato is dependent on their transduction and in its absence PR-expressing neurons may go undetected. This was evident in the wide variability in the volume of EC, CA1 and DG labeled with tdTomato fluorescence.

Sex differences exist for various cognitive tasks; women perform better in episodic memory tasks, whereas men have an advantage in visuospatial tasks^59,60^. However, the role of progesterone-PR signaling is shared in males and females, which is distinct from the often opposing actions of PR signaling in the mating behavior of males and females^61^. Correct identification of an object/individual depends on properly integrated sensory cues. PRs may regulate the generation and/or integration of sensory cues, and their deletion could affect these functions. Although not typically thought of as reproductive behavior, spatial and recognition memories are also critical for successful reproduction. Since, the home range of males (462 m^2^) is double that of females (214 m^2^)^62^, spatial memory and object identification could be more critical in them than in females. Sexual selection favors mating of individuals with better traits; a better spatial memory could help territory marking by males, as seen in lekking hummingbirds^63^. Females also appear to prefer males with a better spatial ability in meadow voles^64^. A better spatial memory could favor sexual selection (larger size) by increasing foraging avenues through a better memory of food source locations. An earlier study found that PRKO males are less aggressive^21^, which raises the possibility that territory marking and defense may be affected. Hence, although reproduction per se does not seem to be affected by PR deletion in males in laboratory conditions, absence of this signaling could be disadvantageous for reproduction in males in real world.

PR expression in the Cajal-Ritzius cells of the DG of developing mice is proposed to regulate the perforant path innervation of the molecular layer, inhibiting PRs with RU486 during the first week of life affects episodic-like memory in adult males^23^. Similarly, PR function in the ventral tagmental area is also critical for cognitive performance in adulthood^22^. The lack of PRs throughout the development of PRKO mice could have affected the EC-DG synapses. However, this effect was not exclusively due to the developmental absence of PRs, since, RU486 treatment in adulthood also impacted novel object recognition. Thus, impairing the EC-DG network activity may be sufficient to alter episodic-like memory formation.

We have previously found that PR activation increases the excitability of entorhinal cortical neurons in adult females^26^. A similar effect of PR activation in male mice predicted that treatment with PR agonist segesterone would increase the number of active neurons in the entorhinal cortical-hippocampal network. Indeed, our experiments using TRAP mice in which activity-dependent promoter cFos drives the expression of tdTomato reporter protein revealed an increased number of active neurons in the hippocampus and entorhinal cortex. Surprisingly, however, the TRAPed neurons in the lateral entorhinal cortex were spread over all the layers in contrast to the predominant expression of PRs in the superficial layers, which indicates that PR agonist treatment likely activated PR-expressing neurons and their target neurons.

In conclusion, the excitatory neurons of EC and hippocampi express PRs and their presence is necessary for regulating episodic-like and spatial reference memories.

## Materials and methods

### Materials

Segesterone, a progesterone receptor antagonist sold under the brand name Nestorone, and other common chemicals were purchased from Sigma-Aldrich.

### Animals

Animals were handled according to a protocol approved by the University of Virginia Animal Care and Use Committee (ACUC), compliant with the ARRIVE guidelines. All methods were performed in accordance with the relevant guidelines and regulations. The mice were anesthetized with isoflurane, decapitated, and the hippocampal and entorhinal cortical tissues were collected for PR mRNA and progesterone level measurements. The animals were also transcardially perfused under isoflurane anesthesia. The mice used for behavior testing were euthanized using a CO2 chamber at the end of the experiments. Mice with a floxed first exon of *Pgr* (PR^fl/fl^) were a kind gift from Dr. M. Luisa Iruela-Arispe (University of California, Los Angeles, CA)^50,65^. In these mice, the 5’ loxP site is approximately 2.1 Kb upstream of the start codon of PR-B isoform and the 3’ site is downstream of the 1st exon. The mice were crossed with nestin-Cre mice (B6.Cg-Tg(Nes-Cre)1Kln/J, The Jackson Laboratory # 003771) that express cre recombinase in neurons and glia to generate brain-specific PR deletion. We and others have used the PR^fl/fl^ mice before to generate a whole-body PR knockout or conditional PR deletion^50,66,67^. The colony was maintained by breeding PR^fl/fl^-cre+ ve male mice (PRKO) with PR^fl/fl^-cre−ve females. This breeding generated PR^fl/fl^-Cre +ve animals (PRKO) and PR^fl/fl^-Cre -ve mice (which we referred as wild-type, WT). Mice expressing Cre recombinase under the control of Pgr (B6.129S(Cg)-Pgrtm1.1(Cre)Shah/AndJ, The Jackson Laboratory #017915) were also used. An advanced version of TRAP mice described in our earlier study was also used^27^. All animals had ad libitum access to food and water and were maintained on a 12-h light and 12-h dark cycle (lights on at 6 AM, lights off at 6 PM). All experiments were performed with 50–70 day-old animals.

### Behavior testing

The behavior tests were performed only in male animals and separate cohorts of animals were used for each of the tests. The animals were handled for three days, five minutes each before behavioral testing.

A cohort of C57Bl6 animals were treated with RU-486 (10 mg/kg, subcutaneous, sc; n = 7), a PR antagonist that should produce similar effects as the PRKO, daily for a week before behavior testing. The behavior was tested a day after the last injection. Control animals were similarly treated with vehicle (20% β-hydroxycyclodextrin, n = 9) and then tested.

#### Novel object recognition test

Non-spatial working memory was evaluated using a novel object recognition task^68^. In this test increased exploration of the novel object indicates that a memory trace for the familiar object was properly encoded. Briefly, the animals were habituated to the arena (45 × 45 × 40 cm, Harvard Apparatus) for 10 min for two days (habituation) before the experiment (Fig. 4A). On the experiment day, a total of 14 WT and 16 PRKO were exposed to the arena (familiarization phase) with two identical objects placed at the opposite corners and allowed to explore for 5 min. The animals were returned to their home cages. After a delay of one hour 7 WT and 10 PRKO mice were re-exposed to the arena (testing phase) for 5 min. During the recognition test, one of the original objects was replaced with a novel object that was similar in size but differed in both color and shape. The remaining mice (7 WT and 6 PRKO) were re-exposed to the arena after 8 h of delay. A video recorder and AnyMaze software (Stoelting) were used to quantify exploration behavior during the familiarization and testing phases. The exploration was quantified in terms of time spent in the target zone (i.e. 2 cm surrounding the objects). Novel object preference was determined as percent time spent with the novel object [i.e. (time with novel object)/(total time spent with the two objects) × 100], where 50% corresponded to a chance exploration. Additionally, discrimination index [(time with novel object-time with familiar object)/total object exploration time] was also determined^32^. A negative discrimination index indicates a preference for the familiar object. The animals were excluded from the experiment if the total object exploration time was less than 20 s during the familiarization phase.

#### Object place assay

The procedure to assess spatial memory discrimination was similar to the novel object recognition test described above. The only deviation occurred during the testing phase, where one of the original objects explored during the familiarization phase was moved to a new location (Fig. 5A). Thus, the novel experience during this test involved the new location of a familiar object. Eight WT and PRKO mice each were used for these studies. There was one hour delay between the familiarization and testing phases during which the animals were kept in their home cages.

#### Y maze forced alteration assay

The animals were exposed to the Y maze apparatus for 10 min with one of the two arms blocked (Fig. 5D). After the first exposure, the animals were returned to the home cage for 30 min and re-exposed to the apparatus for another 10 min with both arms open. The number of entries into novel versus familiar arms and the percentage of time spent in the novel versus familiar arms were compared among animals. If previous experience with a familiar arm is encoded into memory, animals tend to spend less time re-exploring the remembered context and show an increase in exploration of the novel addition to the testing arena. Six animals of each genotype were used in these studies.

#### Contextual fear conditioning

A chamber (27 × 21 × 21 cm; Med Associates Inc) equipped with an infrared camera at the front for recording the behavior was used for contextual fear conditioning. The chamber floor consisted of stainless-steel rods placed 6 mm apart. A standalone aversive stimulator applied foot shock via the floor rods. An audio generator–connected speaker at the chamber top provided an auditory signal, with a background noise of 60 dB supplied by a fan at the side of the chamber. The chamber was cleaned with 70% alcohol in between animals and wiped with 1% acetic acid before the animal exposure. For conditioning, the animals were placed in the chamber and allowed to explore for 3 min before the application of five 2-s foot shocks paired with a tone (90 Db, 5000 Hz, 20 s). The shock (0.5 mA for 2 s) was contiguous with the last 2 s of the tone. Each tone-shock stimulus was separated by 60 s. The animals were removed from the chamber 2 min after the last shock and returned to the home cage. Video Freeze software was used to track the animal and to apply tone and foot shock. Six WT and 7 PRKO mice were used in these studies.

We tested context recall 24 h after conditioning. After cleaning the chamber with 1% acetic acid, the animal was placed in it for 8 min without tone or shock. Freezing was defined as immobility for 2 s, and the freezing time was expressed as a percent of total time in the chamber.

#### Morris water maze (MWM) task

The MWM test was conducted in a circular opaque milky water-filled tank (6 ft wide and 3 feet deep) with the escape platform (in NE quadrant) in a room with visual spatial cues, which remained constant throughout the experiment^69^. Mice (n = 8 each of PRKO and WT) were trained for 5 days in four 1 min-trials separated by 15 min and their movement was recorded with a video tracking system (Noldus Information Technology, Leesburg, VA). Mice were placed in the pool from one of the four start locations (i.e., north, south, east, west) during training. On the first two days, if the animals did not find the escape platform at the end of one min, they were gently guided to the platform and kept on it for 15 s. On the day of testing, the platform was removed and a single probe trial of 1 min was performed with the entry point SE for all the animals. Ethovision 13 software was used for data acquisition and analysis. The latency to reach the platform during training sessions and the time spent in the target quadrant during the probe trial on day 6 were compared between PRKO and WT mice.

### Estrous cycle

The vaginal cytology of female mice was evaluated daily between 9 to 10:30 AM to determine the stage of the cycle as described previously^50^.

#### qRT-PCR

**Figure 8:**
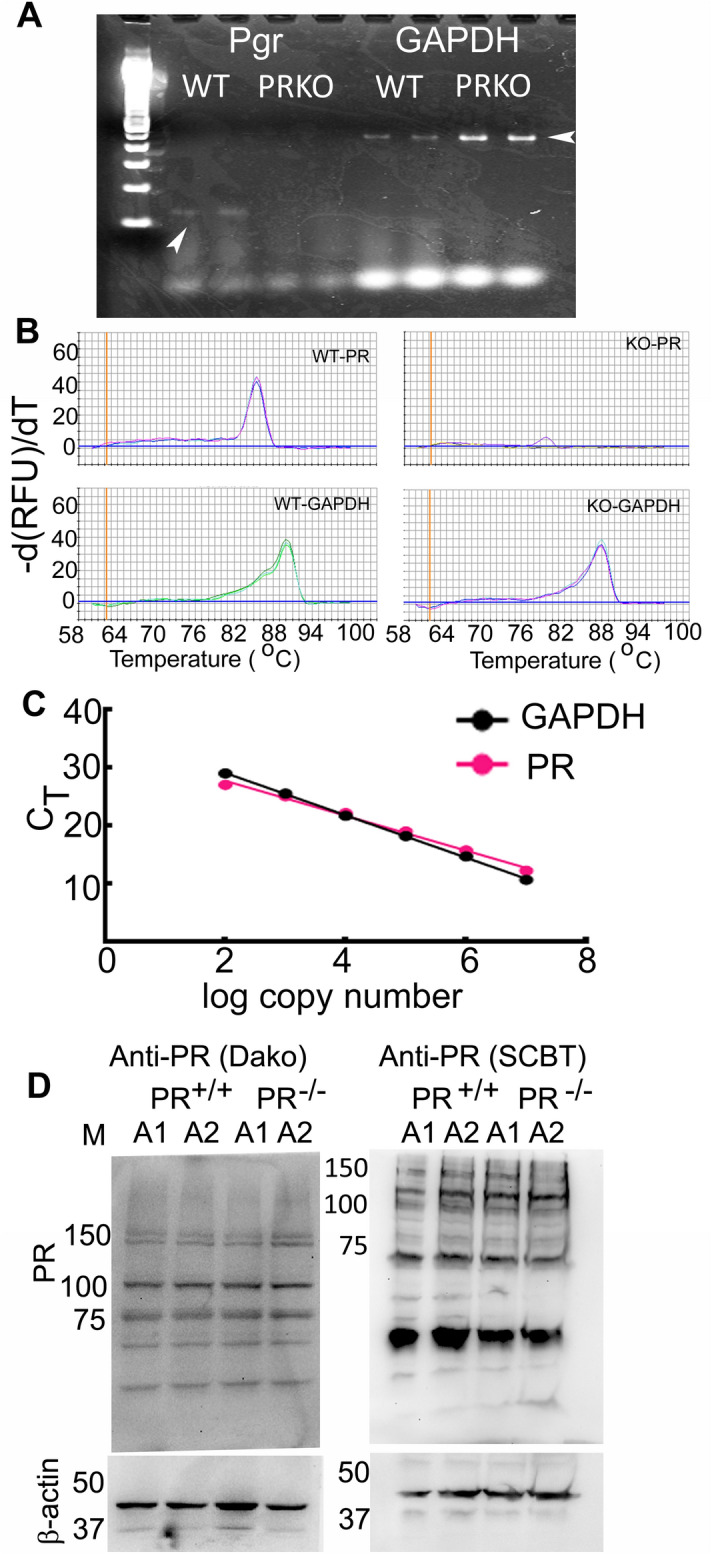
Validation of the qRT-PCR assay. (**A**) A PCR was performed using primers amplifying a fragment of PR and GAPDH using cDNA as a template. The PCR reaction generated a single band of the expected size in the WT animals; an amplification product was not seen in the PRKO animals. (**B**) Melting curves of PR and GAPDH qPCR from representative PRKO and WT mice. (**C**) Efficacy of amplification of PR and GAPDH primers. (**D**) Western blots of brain proteins using two anti-PR antibodies. The expression of β-actin was used as a loading control. Molecular weight markers are shown in lane M. Please note that the antibodies reacted with multiple proteins suggestive of lower specificity. The β-actin blot is cropped to show only the signal, full blot is included as a supplementary file.

Hippocampus and EC were isolated from male and gonadally-intact female mice in the estrus stage of the cycle. After one complete estrous cycle was monitored, the tissue was harvested in estrus stage of the 2nd cycle. Uterine tissue of female mice in the estrus stage was used as a control for normalization. The mice were anesthetized under isoflurane anesthesia, then decapitated and the regions of interest were microdissected from the brain or body. Total RNA isolation and cDNA synthesis was performed as described before^50^. The qPCR assay was performed using sensiFAST SYBR and fluorescein kits (BioLine) and primers PGR-F: TCTACCCGCCATACCTTAACT and PGR-R: GTGACAGCCAGATGCTTCAT and GAPDH-F: ACAGTCCATGCCATCACTGCC and GAPDH-R: GCCTGCTTCACCACCTTCTTG. The gene expression was determined using the 2^−ΔΔCt^ method, with the expression in the uterine tissue taken as a control for normalization. The PCR cycle consisted of 95 °C for 5 min, and 40 cycles of 95 °C for 15 s followed by 60 °C for 15 s. Negative controls included reactions without RT. The validation of the qRT-PCR assay is provided in Fig. 8. The PCR amplicon was sequenced to confirm the amplification of the correct target. To validate the 2^−ΔΔCt^ method, Ct values for PR and GAPDH were determined for cDNA dilutions ranging from 1:10 to 1:100,000. The log cDNA dilution was plotted against ΔCt; the absolute slope of the line was 0.03, indicating that the efficiency of amplification of PR and GAPDH was similar over a wide range of copy numbers. The C_T_ versus log copy number plots were comparable for the PR and GAPDH PCRs (Fig. 8C), and the amplification product was absent in the PRKO animals (Fig. 8A, B).

#### TRAPing of active neurons

TRAP2 male mice were housed four per cage. In these double transgenic mice, a Cre recombinase tagged with a (mutated) estrogen receptor (ER, that does not bind to the endogenous ligand) is expressed under the control of cFos promoter. A tdTomato with an upstream stop codon flanked by the loxP site is present in the rosa locus. Administration of a synthetic estrogen receptor (ER) ligand 4-hydroxytamoxifen (4-OHT) in these mice induces translocation of the Cre-ER into the nucleus of cells with an active cFos promoter. The subsequent removal of the stop codon permits tdTomato expression under the regulation of CAG promoter. This strategy allows tdTomato labeling of neurons active during 60–90 min prior to 4OHT administration. Segesterone (10 mg/kg, subcutaneous, sc, a single dose) or vehicle (20% β-hydroxycyclodextrin) were administered to the mice, and two days later, 4-OHT (50 mg/kg, sc) was administered. The cage was kept in a quiet area, and the mice were not handled on that day before the 4-OHT injection to avoid neuronal activation due to other stimuli. Seven days after 4-OHT administration, the animals were transcardially perfused under isoflurane anesthesia with a solution containing 4% paraformaldehyde and 1% acrylamide and post-fixed in the same overnight. As described before, two hundred micron-thick horizontal brain sections were processed for passive tissue clearing^70^. The expression of neuronal marker protein NeuN (anti-NeuN, 1:200, MAB377, Millipore) was determined to identify neuronal tdTomato expression. Alexa Fluor™ Plus 488-labeled anti-mouse secondary antibody (1:1000, ThermoFisher Scientific) was used to detect the primary antibody.

#### Image acquisition and analysis

The tissue was imaged using a Nikon Eclipse Ti-U microscope equipped with a Nikon confocal C2 scanner, DU3 High Sensitivity Detector System, and Nikon LUN4 4 Line Solid State Laser System under 10× 0.45 numerical aperture lens with 512 × 512 frame size. Ten µm optical sections were acquired, and images were tiled and stitched using NIS-Elements software. The images were processed and analyzed using Imaris 8.3.1 software (Bitplane Scientific, Zurich, Switzerland). The regions of interest were marked using Paxinos Atlas.

#### Measurement of progesterone levels

Combined EC and Hp tissue from WT and PRKO male mice was isolated, snap-frozen, and stored at − 80 °C until use. The steroid levels were measured using the DetectX progesterone enzyme immunoassay kit (Arbor Assays, Ann Arbor, MI) with a 50–3200 pg/mL detection range. The steroids were extracted from the tissue with acetonitrile using a steroid tissue extraction protocol (Arbor Assays). The steroids were suspended in 50 µl ethanol, and 10 µl of the suspension was used for the assay. All the samples were run in duplicate.

#### PR deletion in the EC

AAV9 expressing GFP-Cre under the control of CamKII promoter (pENN.AAV.CamKII.HI.GFP-Cre.WPRE.SV40, Addgene #105551) was used to delete the expression of PRs in the EC of adult PRfl/fl males. Control mice were injected with AAV9 expressing GFP under the control of CamKII promoter (pENN.AAV.CamKII0.4.eGFP.WPRE.rBG, Addgene # 105541). The injections were performed bilaterally. The stereotaxic coordinates used for viral injections were anteroposterior [AP] − 4.6; mediolateral [ML], −/+ 3.0; dorsoventral [DV], − 3.7 and-3.0 from dura for central and medial EC and AP − 3.7, ML −/+ 4.2, and DV − 3.5 and − 3.0 from dura for lateral EC. A Hamilton Company (Reno, NV) syringe (Hamilton 7000 Glass, 1 μl, 0.3302 mm) was loaded with virus solution and mounted in the peristaltic pump holder (Harvard Apparatus, Holliston, MA; P-1500), 200 nl of the virus was injected at each site at a flow rate of 200 nl/min. The experiments were performed two weeks after viral injection. At the end of the experiment, viral transduction of the EC cortical neurons was confirmed by evaluating GFP expression in the brain slices.

### Characterization of PR expression using *pgr*-cre mice

AAV9 expressing a flex tdTomato (pAAV-FLEX-tdTomato, Addgene # 28306) was used to evaluate PR expression in the EC-hippocampal network in the Pgr-Cre mice. A 100 nl of the virus was injected unilaterally in the medial/central EC and lateral EC (please see the stereotaxic coordinates above), in the DG (AP − 3.6, ML 2.5, DV − 2.3), and the CA1 (AP − 3.0, ML 3.5, DV − 3.5); the animals were perfused 14 days after virus injection, 40 μm-thick sections were prepared and co-labeled with NeuN using standard immunohistochemistry^71^. The sections were also immunolabeled with anti-CamKII (1:500, ab34073, Abcam) to determine whether the principal neurons expressed PRs. The sections were labeled with anti-GluA2 (1:1000, clone 6C4, Millipore), anti-somatostatin (1:500, ab108456, Abcam), and anti-parvalbumin (1:500, ab427, Abcam) antibodies using standard protocols to evaluate PR expression in the interneurons and hilar mossy cells. Alexa Fluor™ Plus 488-labeled secondary antibody was used to detect the bound primary antibody. To quantify the PR expression, the outline of the region of interest (ROI) was marked and the tdTomato fluorescence was quantified using Imaris software. The fluorescence was normalized to the NeuN fluorescence in the ROI. To quantitatively assess PR expression in the GABAergic vs glutamatergic neurons, the colocalization function in the Imaris software was used. A colocalized channel was built using the overlap of tdTomato fluorescence with that of PV, Som, CamKII or GluA2 immunoreactivity. The number of colocalized neurons were counted using the “spots” function in the software. The number of colocalized neurons was normalized to the total number of Tdtomato-expressing neurons.

### Statistical analysis

Graphpad Prism 9 was used to perform statistical comparisons. All the values are expressed as mean ± standard deviation. The differences were considered significant when the *p* value was less than 0.05. The normal distribution of the data was determined using the Kolmogorov–Smirnov test. The data were compared using the student's t-test, one sample t-test, ordinary one-way ANOVA followed by Šídák's or Tukey’s multiple comparisons tests, or two-way repeated measures ANOVA.

## Supplementary Information

Supplementary Figure 1.

## Data Availability

The datasets generated and analyzed in this work are available from the corresponding author upon reasonable request.
