# Supplementary figures and images for: Limbic progesterone receptors regulate spatial memory
Source: Sci Rep. 2023 Feb 7;13:2164. doi: 10.1038/s41598-023-29100-2 (PMC9905062; doi:10.1038/s41598-023-29100-2)

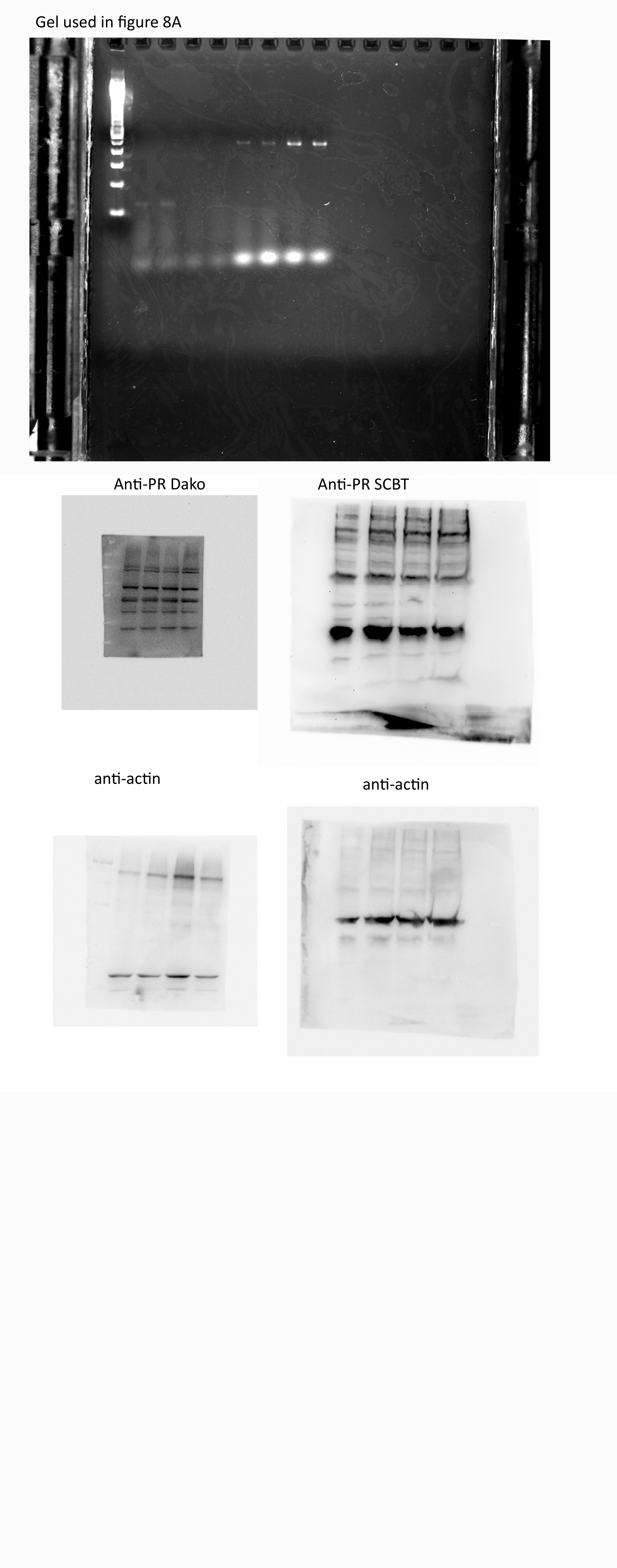

Supplement: Supplementary file 1 — Supplementary Figure 1. [file 41598_2023_29100_MOESM1_ESM.tif]
